# Addressing field data scarcity in algal bloom surveillance: integrating fuzzy inference and orbital remote sensing in Brazilian reservoirs

**DOI:** 10.1007/s11356-026-38094-z

**Published:** 2026-07-30

**Authors:** Ebenézer de Paula Carvalho, Nilo Antonio de Souza Sampaio, Hugo Pimentel Tavares, Carin von Mühlen

**Affiliations:** DEQA Department of Chemistry and Environmental, UERJ FAT: Universidade do Estado do Rio de Janeiro Faculdade de Tecnologia, Resende, Brazil

**Keywords:** Eutrophication, Remote sensing, Algal blooms, Fuzzy logic, Sentinel-2, Water quality, Trophic State Index

## Abstract

Chronic scarcity of continuous in situ sampling severely hampers trophic monitoring in tropical reservoirs, restricting the application of data-intensive models in regulatory environments. Therefore, the aim of this study was to develop a cross-validation framework integrating a Mamdani Fuzzy Inference System (FIS) with Sentinel-2 Normalized Difference Chlorophyll Index (NDCI) retrievals via Google Earth Engine (GEE) to overcome these limitations. The methods involved parameterizing the FIS with subtropical thresholds using a 16-year limnological dataset from the Billings Reservoir (Brazil). This system was then cross-validated against satellite-derived NDCI distributions using non-parametric statistical tests. Subsequently, it was applied to the Funil Reservoir to evaluate spatial transferability based exclusively on NDCI mapping, without concurrent in situ validation. The results demonstrated a statistically significant validation at the Billings Reservoir (Kruskal-Wallis, p<0.05), confirming that NDCI medians vary systematically across fuzzy-classified trophic states. Additionally, Levene’s test revealed homogeneous variance for total phosphorus (TP) (W=3.09, p=0.051) contrasting with heteroscedastic chlorophyll-*a* (Chl-*a*) (p<0.05), characterizing the distinct dispersion regimes of eutrophication drivers and biological responses. Furthermore, the NDCI-based application to the Funil Reservoir yielded synoptic maps that indicated potential algal bloom expansion at resolutions unattainable by conventional monitoring. In conclusion, this approach establishes remote sensing-based surveillance via NDCI, initially calibrated by fuzzy logic, as a valuable screening tool for eutrophication monitoring in data-scarce aquatic systems.

## Introduction

The eutrophication of continental water bodies, driven by nutrient inputs from sewage, industrial effluents, and agricultural runoff, constitutes one of the most critical drivers of water quality degradation globally. In tropical reservoirs, elevated temperatures and high nutrient availability favor cyanobacterial blooms, which pose urgent risks to public health through the production of toxic secondary metabolites called cyanotoxins. Managing these outbreaks is technically complex: inadequate treatment processes can trigger cell lysis, releasing otherwise intracellular toxins into the water supply, where they resist conventional removal and bioaccumulate through the trophic chain. Addressing these complex challenges and formulating effective policies requires integrating economic, environmental, and social approaches to reconcile water use efficiency with the sustainability of water systems (de Carvalho et al. 2026).

The inherent temporal variability of biological systems exacerbates this scenario: cyanobacterial density can fluctuate from safe levels to conditions of extreme risk within a few weeks, driven by climatic pressures such as precipitation and temperature. Consequently, rigid and widely spaced sampling schedules may fail to detect biomass peaks, generating information gaps that compromise water security (Haakonsson et al. 2024).

The lack of consistent data resulting from these limitations creates a vicious cycle in water resource management (Zimmermann 2024). In many systems, the monitoring routine is not fully met, rendering the number of available data regarding critical biological parameters such as cyanobacteria and Chl-*a* negligible. This data scarcity, or the presence of censored, edited, or deliberately removed data, hinders the calibration of predictive models and the application of decision-support tools, such as machine learning models. While these algorithms are increasingly recognized for enabling objective and accurate water quality assessments (Soltani et al. 2025), they inherently depend on robust historical series to reduce uncertainties. Therefore, the exclusive reliance on in situ methods results in lagged monitoring, often incapable of providing the early warnings necessary for the timely mitigation of sanitary risks.

Given the intrinsic limitations of conventional monitoring, orbital remote sensing emerges as a structural component for large-scale surveillance (Adjovu et al. 2023). However, translating satellite radiance into Chl-*a* concentrations in optically complex inland waters (Case 2) is challenging, as traditional blue/green ratio algorithms fail due to spectral overlap with colored dissolved organic matter (CDOM) and non-algal suspended sediments. To overcome this, this study employed the Normalized Difference Chlorophyll Index (NDCI) via the *Sentinel-2* MultiSpectral Instrument (MSI). By targeting the Chl-*a* absorption feature near 665 nm and the reflectance peak near 705 nm, the NDCI effectively isolates the phytoplankton scattering signal, rendering it highly stable in turbid, productive reservoirs.

While the NDCI successfully resolves the optical challenges of biomass retrieval, translating these spatial observations into regulatory trophic classifications under chronic data scarcity requires specialized modeling. Therefore, we coupled the orbital data with a Mamdani Fuzzy Inference System (FIS). This approach offers a distinct advantage over classical machine learning classifiers, such as random forest or support vector machines, which require large, balanced datasets across seasonal cycles to avoid overfitting. In contrast, the FIS translates established environmental thresholds and expert knowledge into heuristic rules (Yang et al. 2026), enabling reliable trophic classification even when dense matchups of coincident in situ and satellite observations are unavailable. Similar hybrid approaches coupling FISs with geospatial datasets have been successfully implemented to assess other complex environmental systems, such as groundwater potential zones (Babu and Maury 2025).

The fundamental advancement of this work is the integration of this expert-calibrated FIS with satellite-derived NDCI distributions to establish a cross-validation framework that operates independently of continuous concurrent in situ sampling. Consequently, this study was designed with two primary objectives: first, to calibrate the Mamdani FIS using a 16-year limnological dataset from the Billings Reservoir (São Paulo, Brazil) and cross-validate its classifications against Sentinel-2 NDCI retrievals, and second, to evaluate the spatial transferability of this framework to the Funil Reservoir (Rio de Janeiro, Brazil) as a screening tool. Ultimately, this work is motivated by the critical need to establish resource-efficient, high-frequency trophic surveillance, providing water resource managers with a scalable protocol to identify potential algal blooms and guide proactive interventions in data-scarce aquatic systems.

## Methods

**Fig. 1:**
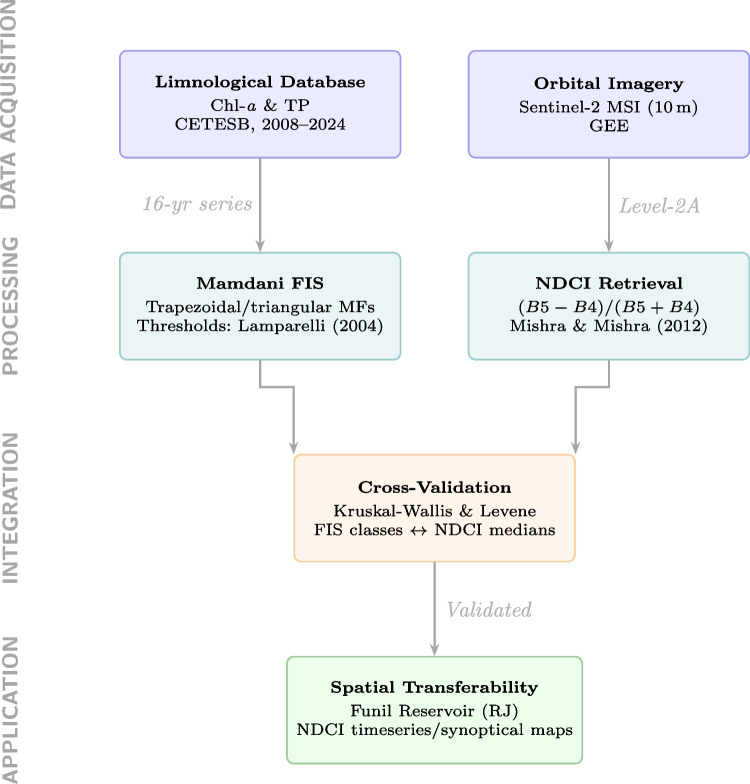
Methodology flow chart of the dual framework integrating in situ fuzzy trophic inference and orbital remote sensing (NDCI) for eutrophication monitoring and transferability to data-scarce reservoirs

The methodological strategy was structured in two sequential stages of analysis and validation, as illustrated in the flow chart presented in Fig. 1. The first stage consisted of developing a FIS for trophic state classification, fed exclusively by historical in situ data of Chl-*a* and TP from CETESB, the Environmental Agency of the State of São Paulo, at the Billings Reservoir in São Paulo, Brazil. In the subsequent stage, the quality classes resulting from the FIS were statistically compared with the NDCI values derived from Sentinel-2 MSI imagery. The objective of this cross-validation was to demonstrate that spectral index variations track the changes in fuzzy trophic classification, thereby validating the NDCI as a consistent proxy for supporting the proposed monitoring framework. Accordingly, this validated index was applied to the Funil Reservoir as an indicative spatial mapping tool, functioning independently of concurrent local in situ validation.

To ensure the consistency of the analyses, the datasets obtained from CETESB were subjected to exploratory data analysis using the Python programming language in conjunction with the Pandas library (Mckinney 2010).

### Study areas

#### Model development: billings reservoir

The Billings Reservoir, located to the southeast of the São Paulo Metropolitan Region (SPMR), represents the largest water storage system in this region, playing a strategic role in local water security (Milz et al. 2022). With a drainage area of approximately 560 to 580 km$^2$, the watershed entirely encompasses the municipality of Rio Grande da Serra and partially overlaps with the municipalities of Diadema, Ribeirão Pires, Santo André, São Bernardo do Campo, and São Paulo (Wengrat and de Campos 2011).

**Fig. 2:**
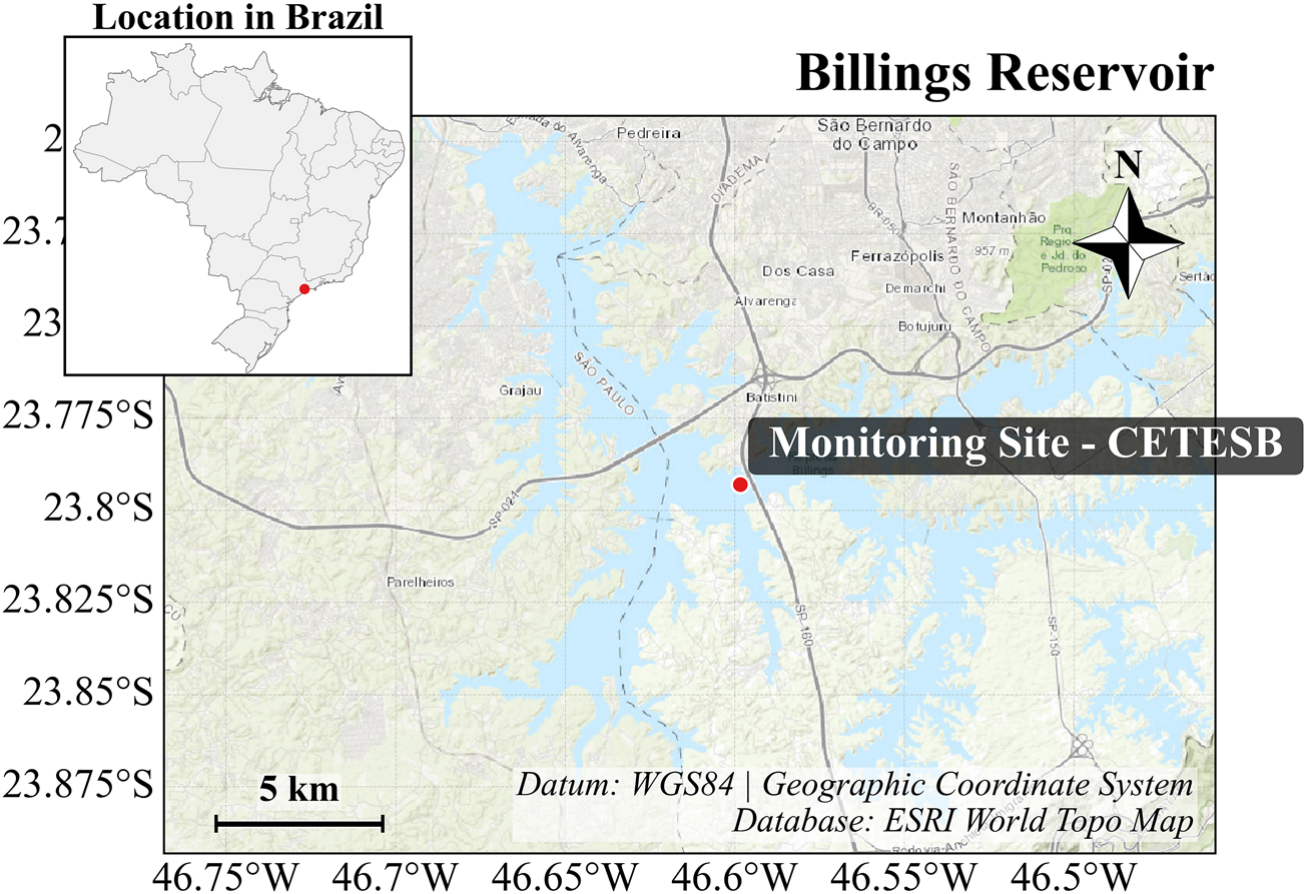
Geographical location and morphological structure of the Billings Reservoir. Source: the author

Morphometrically, the reservoir exhibits a highly complex dendritic pattern, characterized by an elongated, narrow central body connected to eight primary branches: Rio Grande, Rio Pequeno, Capivari, Pedra Branca, Taquacetuba, Bororé, Cocaia, and Alvarenga (Wengrat and de Campos 2011). Its maximum flooded area extends to approximately 127 km$^2$, containing a total volume of approximately 1.2×10$^9$ m$^3$ and reaching a maximum depth of 18 to 19 m (Milz et al. 2022; Wengrat and de Campos 2011). The geographical configuration of the reservoir is presented in Fig. 2.

**Fig. 3:**
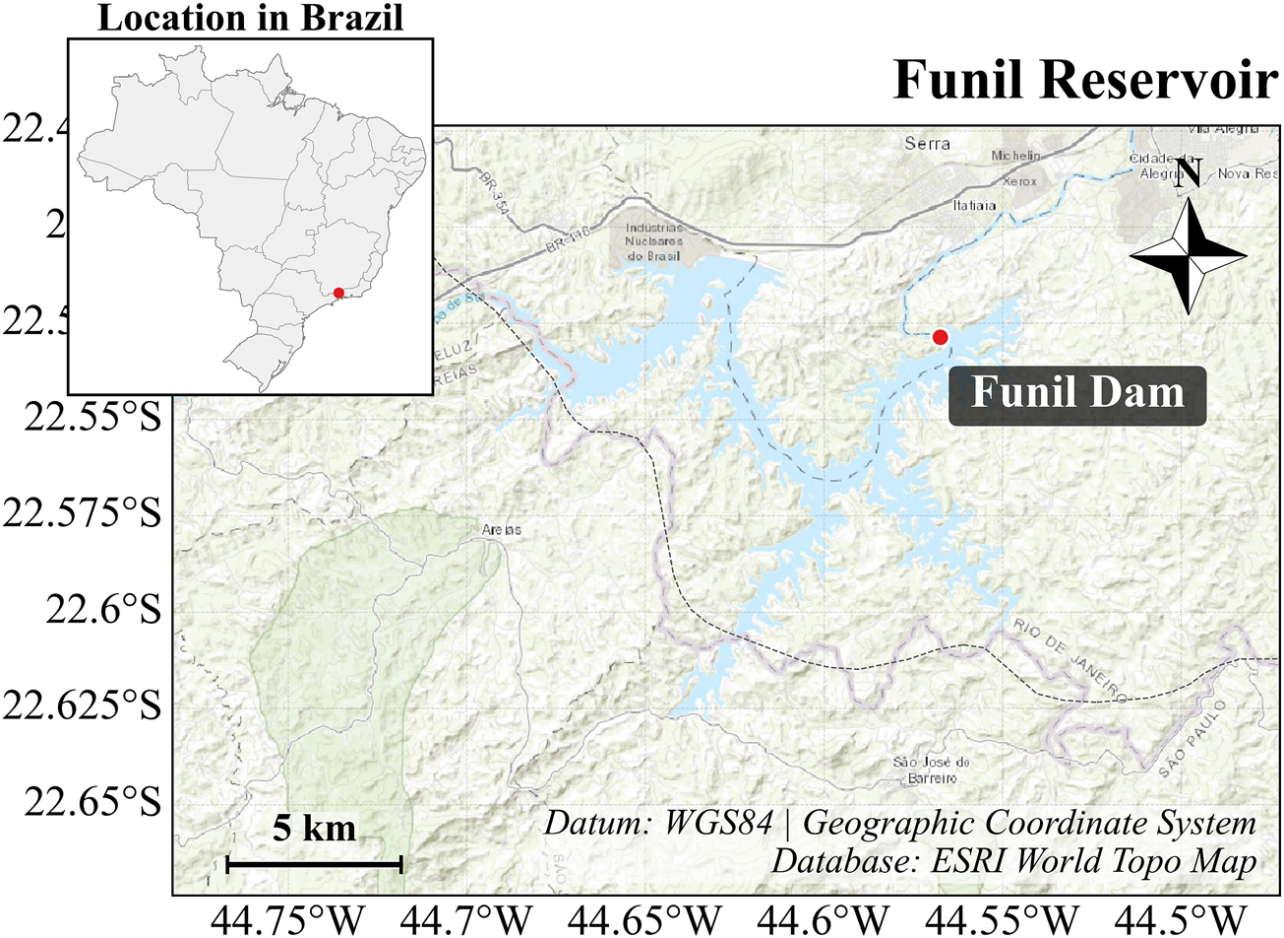
Geographical location and spatial morphology of the Funil Reservoir. Source: the author

#### Application case: the Funil Reservoir

To evaluate the operational transferability of the NDCI as an indicative mapping tool, the Funil Reservoir (22$^\circ$ 30′ S; 44$^\circ$ 45′ W) was selected as the field application case. Because this case study demonstrates the framework’s utility for unmonitored environments, the Funil analysis proceeded without concurrent local in situ validation. This selection was primarily driven by its trophic similarities to the Billings Reservoir, offering a fundamentally comparable yet geographically distinct application scenario. Both reservoirs are located in the Brazilian Southeast region, sharing identical rainfall regimes and temperature seasonality. Furthermore, their nutrient input dynamics are remarkably similar: while Billings receives intense anthropogenic loads from its immediate urban surroundings, Funil receives significant agricultural and urban pollution (primarily nitrogen and phosphorus) transported by the Paraíba River from highly populated areas in the state of São Paulo. This direct transferability framework would not be feasible across geographically distant regions, where substantial variations in precipitation patterns, hydraulic retention times, and land use would confound the optical and trophic relationships. Therefore, their relative regional proximity justifies this comparative analysis.

Situated in the middle reach of the Paraíba do Sul River, the reservoir encompasses the municipalities of Resende and Itatiaia in the state of Rio de Janeiro, projecting into São José do Barreiro, Queluz, and Areias in the state of São Paulo (Ferrão-Filho et al. 2009; de Souza Lima et al. 2016). Inaugurated in 1969, the impoundment operates with a double-curvature arch dam, featuring a maximum height of 85 m and a crest elevation of 468 m. The system maintains a flooded area of approximately 40 km$^2$, a total volume of 8.9 × 10$^9$ m$^3$, and a hydraulic residence time oscillating between 10 and 55 days, highly dependent on seasonal precipitation and operational constraints. The spatial distribution of the reservoir is illustrated in Fig. 3.

The reservoir’s contributing watershed extends over approximately 16,800 km$^2$, predominantly within São Paulo territory. This geographical configuration dictates the water quality dynamics, as the reservoir receives drainage from the portion of the Paraíba Valley within the state of São Paulo, one of the most industrialized and urbanized regions in Brazil. Land use and occupation within the watershed are characterized by a preponderance of pastures, accounting for roughly 60% of the area, followed by forest fragments and reforestation zones. Although urban centers occupy a comparatively small percentage of the land cover, they exert disproportionate environmental pressure due to continuous point-source effluent discharges (de Souza Lima et al. 2016).

**Table 1:** Descriptive statistics of Chl-*a*and TP concentrations (μg/L) in the Billings Reservoir

Statistic	Chl-*a* (μg/L)	TP (μg/L)
*N* (samples)	88	101
Mean	67.13	83.92
Standard deviation	58.26	80.08
Minimum	13.21	7.00
25th percentile	35.91	40.00
Median	48.11	70.00
75th percentile	82.75	100.00
Maximum	416.99	610.00

Source: Elaborated by the author based on CETESB data (2025)

Geomorphologically, the reservoir displays a dendritic configuration, featuring a primary channel following the historical Paraíba do Sul riverbed, coupled with lateral branches formed by tributaries (Ferrão-Filho et al. 2009). This morphological structure, combined with significant depth variations, averaging 22 m and reaching a maximum of 70 m near the dam, promotes the spatial compartmentalization of the water body. Consequently, pronounced longitudinal gradients of water quality and sedimentation are established along the aquatic system (Soares et al. 2012).

### In situ data acquisition and descriptive analysis

To guarantee the reproducibility and robustness of the analyses, the raw input data were initially subjected to an exploratory statistical analysis. Understanding the descriptive statistics is vital to comprehend the dispersion, central tendency, and extreme variability of water quality parameter concentrations during the study period. Table 1 presents a detailed summary of the in situ measurements for Chl-*a* and TP acquired from the CETESB monitoring network.

While these descriptive statistics establish a solid baseline of limnological variability, relying solely on discrete values inherently oversimplifies the ecological risk assessment. The observed difference in sample size (N=88 for Chl-*a* and N=101 for TP) is attributed to operational variations in historical sampling campaigns. However, the FIS modeling was exclusively parameterized using the 88 synchronous paired observations. Unlike data-intensive machine learning algorithms, which require vast datasets to train synaptic weights, fuzzy logic relies on the heuristic encoding of expert knowledge into mathematical rules. Consequently, the paired sample size is statistically sufficient and reliable for this approach, as FIS models are intrinsically designed to handle limited datasets and environmental uncertainty effectively (Gharibi et al. 2012; Yang et al. 2026; Bellini et al. 2025). Recognizing the continuous and highly non-linear nature of eutrophication gradients in tropical reservoirs, this historical dataset provides the essential empirical foundation for the subsequent development of a specialized fuzzy inference framework, capable of mathematically resolving complex biological transitions.

### Trophic classification system and fuzzy inference

The application of a FIS requires defining thresholds adapted to local conditions. As proposed by Lamparelli (2004), Table 2 presents the limnological reference values utilized for the parameterization of the Fuzzy system’s membership functions.

The FIS was implemented using the *scikit-fuzzy* library (Warner et al. 2024) in Python 3.12, following the Mamdani architecture (Bellini et al. 2025; Putri and Saputro 2021), which has been widely adopted in modern environmental models to handle system uncertainties. The input variables selected were Chl-*a* (0–150 μg/L) and TP (0–300 μg/L) (Gharibi et al. 2012; Tundisi and Matsumura-Tundisi 2008). Membership functions were modeled using trapezoidal shapes at the extremities (oligotrophic and hypereutrophic) and triangular shapes for the intermediate classes (mesotrophic, eutrophic, and supereutrophic) to ensure smooth ecological transitions (Gharibi et al. 2012; Putri and Saputro 2021). The membership functions and their detailed parameter coordinates are presented in Table 3.

**Table 2:** Trophic State Index (TSI) and equivalency with total phosphorus, chlorophyll-*a*, and transparency measurements for reservoirs

Trophic level	Total phosphorus	Chlorophyll-*a*	Secchi depth	TSI
	(mg/L)	(μg/L)	(m)	
Ultraoligotrophic	≤0.008	≤1.17	≥2.4	≤47
Oligotrophic	$0.008 < \text {TP} \le 0.019$	$1.17 < \text {Chl} \le 3.24$	$2.4 > \text {D} \ge 1.7$	$47 < \text {TSI} \le 52$
Mesotrophic	$0.019 < \text {TP} \le 0.052$	$3.24 < \text {Chl} \le 11.03$	$1.7 > \text {D} \ge 1.1$	$52 < \text {TSI} \le 59$
Eutrophic	$0.052 < \text {TP} \le 0.120$	$11.03 < \text {Chl} \le 30.55$	$1.1 > \text {D} \ge 0.8$	$59 < \text {TSI} \le 63$
Supereutrophic	$0.120 < \text {TP} \le 0.233$	$30.55 < \text {Chl} \le 69.05$	$0.8 > \text {D} \ge 0.6$	$63 < \text {TSI} \le 67$
Hypereutrophic	>0.233	>69.05	<0.6	>67

Source: Adapted from Lamparelli (2004)

**Table 3:** Parameterization of the membership functions for the Fuzzy Inference System variables

Variable	Trophic class	Type	Parameters
Chlorophyll-*a*	Oligotrophic	Trapezoidal	[0; 0; 1.5; 3.5]
(μg/L)	Mesotrophic	Triangular	[1.5; 7; 20]
	Eutrophic	Triangular	[11; 30; 50]
	Supereutrophic	Triangular	[30; 50; 80]
	Hypereutrophic	Trapezoidal	[60; 80; 150; 150]
Total Phosphorus	Oligotrophic	Trapezoidal	[0; 0; 10; 20]
(μg/L)	Mesotrophic	Triangular	[15; 35; 60]
	Eutrophic	Triangular	[50; 90; 130]
	Supereutrophic	Triangular	[120; 180; 240]
	Hypereutrophic	Trapezoidal	[230; 250; 300; 300]
TSI Score	Oligotrophic	Triangular	[0; 0; 25]
(Output)	Mesotrophic	Triangular	[15; 35; 55]
	Eutrophic	Triangular	[45; 60; 75]
	Supereutrophic	Triangular	[65; 80; 95]
	Hypereutrophic	Triangular	[85; 100; 100]

Note: Trapezoidal parameters [*a*; *b*; *c*; *d*] define the bottom-left, top-left, top-right, and bottom-right vertices. Triangular parameters [*a*; *b*; *c*] define the left vertex, peak, and right vertex. Source: Elaborated by the author based on Lamparelli 2004

**Fig. 4:**
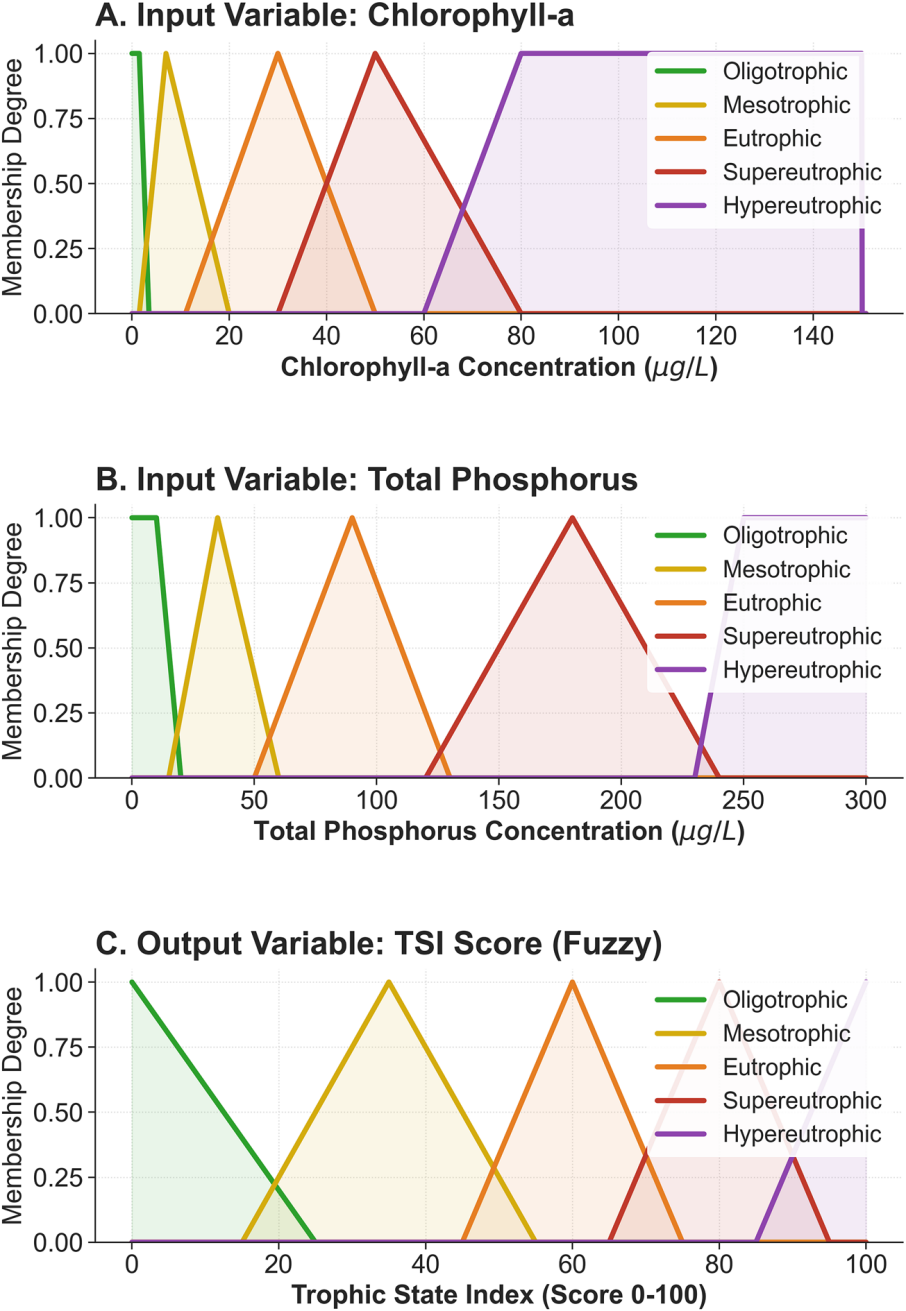
Membership functions of the FIS: A Chl-*a*, B TP, and C TSI Score. Source: Elaborated by the author

The system’s output variable, *Trophic State Index (TSI) Score*, was defined across a normalized universe of discourse from 0 to 100. Figure 4 presents the configuration of the membership functions for the input and output variables.

### Model application and trophic classification

Fuzzy inference was applied to the historical CETESB dataset. For each pair of input values, the system evaluates the membership degrees, activates the corresponding rules, and aggregates the consequents via the Maximum operator. Values exceeding the maximal discourse limits are truncated, ensuring numerical stability while classifying extreme nutrient and biomass concentrations into the highest trophic status, where saturation effects often dominate the relationship between nutrients and Chl-*a* (Gholizadeh et al. 2016; Huisman et al. 2018). Defuzzification was executed using the centroid method to generate the continuous *TSI Score* (Gharibi et al. 2012; Putri and Saputro 2021).

The inference rule base establishes the relational dynamics between the antecedents and the consequent output. The ten implemented rules, incorporating logical AND and OR operators (Bellini et al. 2025; Putri and Saputro 2021), are detailed in Table 4. The rule base was constructed utilizing logical AND operators for lower-tier trophic ranges in a conservative approach and logical OR operators for upper-tier states, supereutrophic and hypereutrophic, ensuring precautionary detection of acute degradation, adhering to the logic of max-min compositions in fuzzy inference networks (Bellini et al. 2025; Putri and Saputro 2021).

### Spectral index retrieval and spatio-temporal synchronization

To evaluate the spatial sensitivity of the model and correlate the in situ fuzzy classification with orbital observations, an experimental multi-point design was established. Four geographic coordinates (designated generically as P1, P2, P3, and P4) were strategically selected adjacent to the primary CETESB monitoring station, distributed within an approximate radius of 500 m. This multi-point approach aligns with spatially distributed sampling strategies commonly adopted in limnological assessments of tropical reservoirs, wherein horizontal heterogeneity is frequently pronounced due to nutrient gradients, variable residence times, and differential tributary influence. Specifically for the Billings Complex, previous studies have successfully employed distributed sampling networks to characterize water quality variability across distinct compartments of the aquatic system (Milz et al. 2022). The spatial replication proposed herein enables the identification of spectral response variations associated with local heterogeneity, including sub-pixel mixing effects, proximity to reservoir margins, and localized biomass concentration gradients.

The orbital image processing pipeline within GEE (Gorelick et al. 2017; Amani et al. 2020) incorporated multiple correction and masking stages to ensure high spectral fidelity. Sentinel-2 MSI Level-2A products were utilized, which undergo Top-of-Atmosphere (TOA) to Bottom-of-Atmosphere (BOA) atmospheric correction at the source using the Sen2Cor algorithm. To mitigate cloud contamination, the Cloud Score+ (CS+) algorithm was implemented using the *cs_cdf* band with a spatial quality threshold of $cs\_cdf \ge 0.60$. This threshold is optimized for aquatic surfaces, where low water reflectance can cause standard algorithms to misclassify water pixels as clouds; thus, a 0.60 cutoff removes opaque clouds and shadows while preserving valid water observations. The water surface was isolated using the Normalized Difference Water Index (NDWI), calculated from the green (B3, ∼560 nm) and near-infrared (B8, ∼842 nm) bands, with a threshold of NDWI≥0.0 to mask land areas. Sun-glint correction was applied by subtracting the shortwave infrared (SWIR) band (B12, ∼2190 nm) from the red (B4) and red-edge (B5) bands. This correction assumes near-complete SWIR absorption by water, meaning any signal recorded in B12 over water arises from specular reflection or atmospheric path radiance, thereby isolating the water-leaving radiance before calculating the NDCI. To address land-water adjacency effects and sub-pixel contamination near the margins, the point coordinates for NDCI retrieval (P1–P4) were strategically placed in the main channels, at distances exceeding 30 m (∼1.5 pixels) from the shoreline. For the generated cartographic outputs, shoreline pixels were dynamically removed by retracting the NDWI water mask using a native GEE non-linear moving minimum filter (*.focal_min(radius=1.5)*) (Kislik et al. 2022). This operation evaluates the neighborhood of each cell, replacing the central value with the lowest local NDWI, effectively eroding the boundary by 1 to 2 pixels and discarding shoreline-contaminated cells.

**Table 4:** Fuzzy Inference System rule base for trophic classification

Rule	Level	Chlorophyll-*a*	Operator	Total phosphorus
R1	Oligotrophic	Oligotrophic	AND	Oligotrophic
R2	Oligotrophic	Oligotrophic	AND	Mesotrophic
R3	Mesotrophic	Mesotrophic	AND	Oligotrophic
R4	Mesotrophic	Mesotrophic	AND	Mesotrophic
R5	Mesotrophic	Mesotrophic	AND	Eutrophic
R6	Eutrophic	Eutrophic	AND	Mesotrophic
R7	Eutrophic	Eutrophic	AND	Eutrophic
R8	Eutrophic	Eutrophic	AND	Supereutrophic
R9	Supereutrophic	Supereutrophic	OR	Supereutrophic
R10	Hypereutrophic	Hypereutrophic	OR	Hypereutrophic

Note: The AND operator mandates that both antecedent conditions are satisfied simultaneously. The OR operator activates the rule when at least one condition is true. The consequent of each rule corresponds to the TSI Score of the class indicated in the Level column. Source: Elaborated by the author

A critical challenge in integrating remote sensing data with historical field campaigns is the latency between satellite overpasses and physical water sampling. To establish a correlation between both datasets, a temporal synchronization protocol was implemented incorporating a tolerance window of ±5 days between the satellite acquisition date and the in situ sampling event, which aligns with the Sentinel-2 orbital revisit period. Given that CETESB’s monitoring campaigns occur at 2-to-3-month intervals, this window represents an operational trade-off: reducing this interval would result in a severe loss of coincident matchups due to cloud cover, preventing statistical validation. This synchronization window maintains sufficient statistical entries while tracking seasonal and regional trophic trends. This temporal merge was computationally executed utilizing the merge_asof function from the Python *pandas* library (Mckinney 2010), ensuring spatio-temporal coherence between the discrete physical parameters quantified by the environmental agency and the continuous optical measurements captured from orbit.

### Statistical distribution and non-parametric validation

Data normality and homoscedasticity across the trophic gradient were rigorously assessed using Shapiro-Wilk and Levene’s tests. Due to the presence of significant non-normal distributions (p<0.001) and heteroscedastic variances inherent to biological bloom responses (in situ Chl-*a* and spectral NDCI), non-parametric methods, specifically the Kruskal-Wallis rank sum test accompanied by Dunn’s post hoc test with Bonferroni correction, were universally employed for variance analysis (Gilbert et al. 2024).

## Results and discussion

### Fuzzy inference outputs and response surface

Table 5 presents a representative sample of the classified dataset, illustrating the assignment process of the *TSI Score* and the corresponding trophic class. Unlike conventional threshold-based indexing (Gholizadeh et al. 2016), our results show that for a constant TP value, variations in the Chl-*a* concentration yield significantly different final scores. This multidimensional sensitivity aligns with the observations of Soares et al. (2012), who demonstrated that physiological responses, such as cyanobacterial biovolume, are driven by complex nutrient-temperature interactions and varying taxa sensitivities rather than static nutrient limitations alone. For instance, samples exhibiting Chl-*a* levels exceeding 100 μg/L reach the maximum designated *TSI Score* of 95, being consistently classified as hypereutrophic. This acute degradation state is consistent with the profound spatial heterogeneity and severe eutrophication previously documented for specific compartments of the Billings Complex by Wengrat and de Campos (2011), and corroborates the mechanistic links between excessive nutrient enrichment and uncontrolled biological proliferation established by Huisman et al. (2018). Conversely, moderate values of both parameters result in intermediate, transitionary classifications.

**Table 5:** Sample of the data classified by the FIS in the Billings Reservoir

Index	Date	Chl-*a*	TP	TSI Score	Trophic
		(μg/L)	(μg/L)		Class
0	2008-01-22 13:30	31.19	50.0	62.71	Eutrophic
1	2008-03-26 14:08	21.92	20.0	60.00	Eutrophic
2	2008-05-14 15:00	40.10	30.0	70.07	Supereutrophic
3	2008-07-10 14:35	24.06	30.0	60.00	Eutrophic
4	2008-09-18 13:50	45.11	20.0	74.03	Supereutrophic
5	2008-11-05 12:17	118.38	30.0	95.00	Hypereutrophic
6	2009-01-29 12:08	38.61	30.0	68.99	Supereutrophic
7	2009-03-26 13:50	43.66	30.0	72.77	Supereutrophic
8	2009-05-28 13:15	37.07	40.0	67.82	Supereutrophic
9	2009-07-30 16:10	101.19	40.0	95.00	Hypereutrophic

Source: Elaborated by the author using CETESB data

**Fig. 5:**
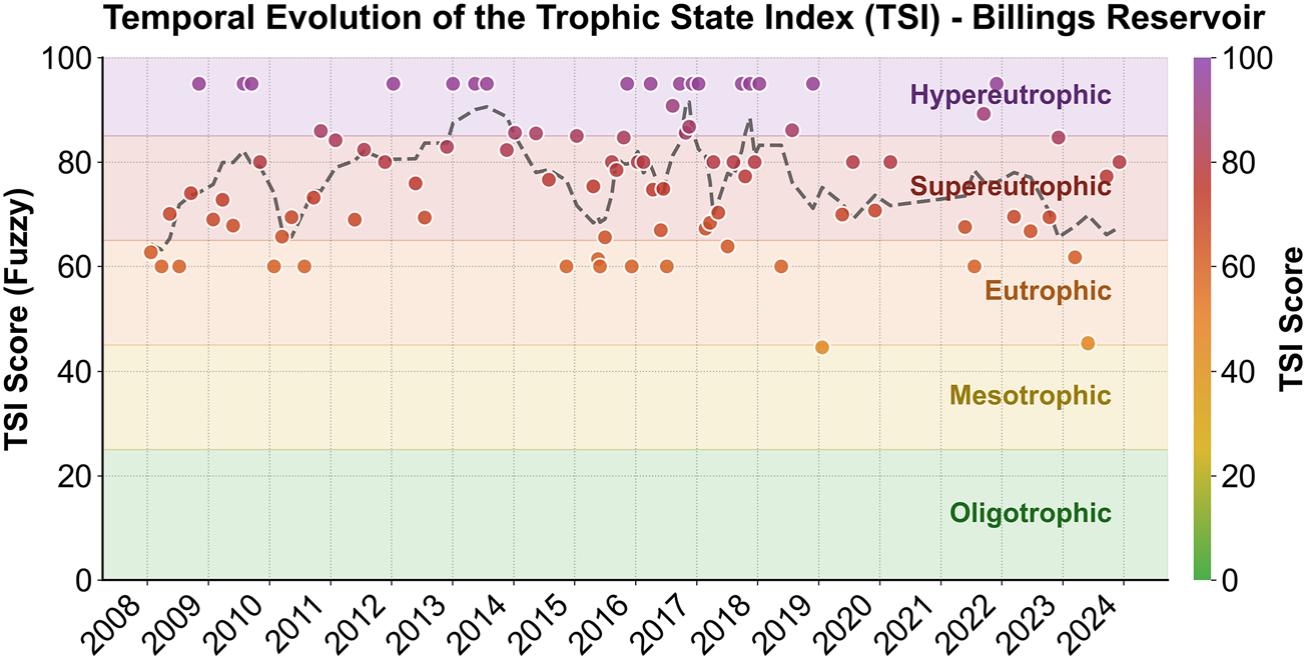
Temporal evolution of the fuzzy-derived TSI in the Billings Reservoir between 2008 and 2024. Individual observations are color-mapped to a continuous trophic gradient, and the dashed line denotes the five-point centered moving average trend

The temporal application of the FIS to the entire CETESB monitoring record yields a continuous diagnostic of the Billings Reservoir’s ecological trajectory. Figure 5 synthesizes this chronological perspective, presenting individual fuzzy-derived *TSI Scores* color-coded according to a graduated trophic palette superimposed with a centralized five-point moving average trend line that filters stochastic sampling fluctuations (Bellini et al. 2025; Gholizadeh et al. 2016). The resulting temporal pattern reveals a scenario of chronic and uninterrupted eutrophication: the overwhelming majority of observations are systematically confined within the supereutrophic (60–80) and hypereutrophic (>80) bands, with no sustained episodes indicative of ecological recovery or trophic regression throughout the 16-year monitoring window. While traditional nutrient loading models typically anticipate seasonal trophic improvements during hydrologically drier periods, the persistence of elevated *TSI Scores* observed here suggests the hypothesis that internal phosphorus recycling from anoxic sediments may operate as a significant endogenous driver. If active, such a mechanism could contribute to perpetuating the eutrophication cycle even during periods of reduced external tributary inflow controls, an effect widely recognized in highly impacted shallow aquatic ecosystems. Furthermore, this temporal stagnation at critical trophic states is consistent with the alternative stable-state framework advanced by Huisman et al. (2018), raising the hypothesis that the reservoir may have breached its natural resilience thresholds and could currently be operating within a potentially self-reinforcing regime dominated by persistent algal blooms and endogenous nutrient feedback loops.

**Fig. 6:**
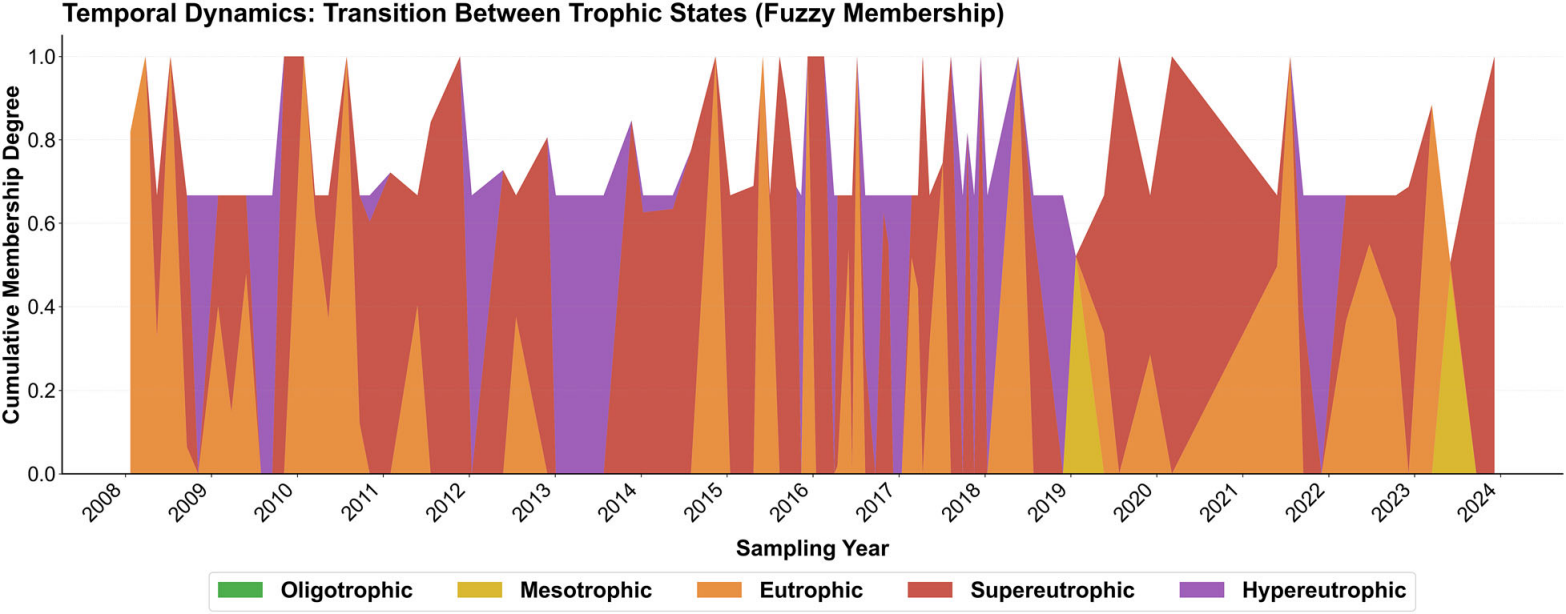
Temporal dynamics of fuzzy membership degrees across the five trophic classes in the Billings Reservoir (2008–2024). The cumulative stackplot illustrates the simultaneous belonging of each observation to oligotrophic, mesotrophic, eutrophic, supereutrophic, and hypereutrophic states

Complementary to the scalar diagnostic, the mathematical decomposition of each observation into its constituent fuzzy membership degrees offers a fundamentally richer perspective on trophic classification uncertainty, a core tenet of fuzzy set theory applied to environmental systems (Gharibi et al. 2012; Yang et al. 2026). Figure 6 presents a cumulative stackplot wherein each temporal observation is vertically partitioned according to its simultaneous belonging to the five trophic classes: oligotrophic, mesotrophic, eutrophic, supereutrophic, and hypereutrophic. This visual representation directly reflects the mathematical output of the membership functions prior to the final defuzzification step, mapping the proportional overlap of ecological states. Unlike traditional rigid boundaries (Gholizadeh et al. 2016), this approach visually captures the fluid ecological transitions within the system. Throughout the 2008–2024 monitoring horizon, the supereutrophic and hypereutrophic fractions collectively dominate the stacked area, with oligotrophic membership remaining virtually absent. This persistent pattern quantitatively reinforces the regime shift diagnosis established in the preceding temporal analysis and corroborates previous spatial evaluations that classified major compartments of the Billings Complex within these upper trophic boundaries (Wengrat and de Campos 2011). Notably, the interval surrounding 2020 exhibits a transient widening of the supereutrophic membership band, an anomaly potentially attributable to the reduced sampling frequency imposed by the operational restrictions during the COVID-19 pandemic, which may have introduced a temporal smoothing artifact in the defuzzified classification output. Nevertheless, the overall visual persistence of elevated membership fractions across the entire series supports the interpretation that the Billings Reservoir has remained locked within a critical state of advanced eutrophication throughout the evaluated historical period, a finding conceptually aligned with recent spatio-temporal water quality assessments of the system’s central body (Milz et al. 2022).

#### Fuzzy system response surface

The three-dimensional visualization of the FIS’s response surface is an essential analytical step to comprehend the non-linear dynamics governing the interactions between the input variables and the model’s categorical output. While classical trophic classification schemes frequently impose arbitrary single-parameter linear thresholds to segregate trophic states (Gholizadeh et al. 2016; Huisman et al. 2018), the continuous geometric mapping presented here argues for a multidimensional continuum. This finding aligns with fuzzy set theory formulations applied to water systems (Gharibi et al. 2012; Yang et al. 2021, 2026), which mathematically establish that biological boundaries are inherently transitional. Furthermore, while de Souza Lima et al. (2016) characterizes similar reservoir dynamics primarily through strict scalar concentrations, the *Z*-matrix computed by our defuzzified simulator corroborates recent advancements demonstrated by Bellini et al. (2025), showing that synergistic limnological interactions-often masked by rigid boundaries-are effectively captured when evaluated as topographic altitudes within the three-dimensional space.

**Fig. 7:**
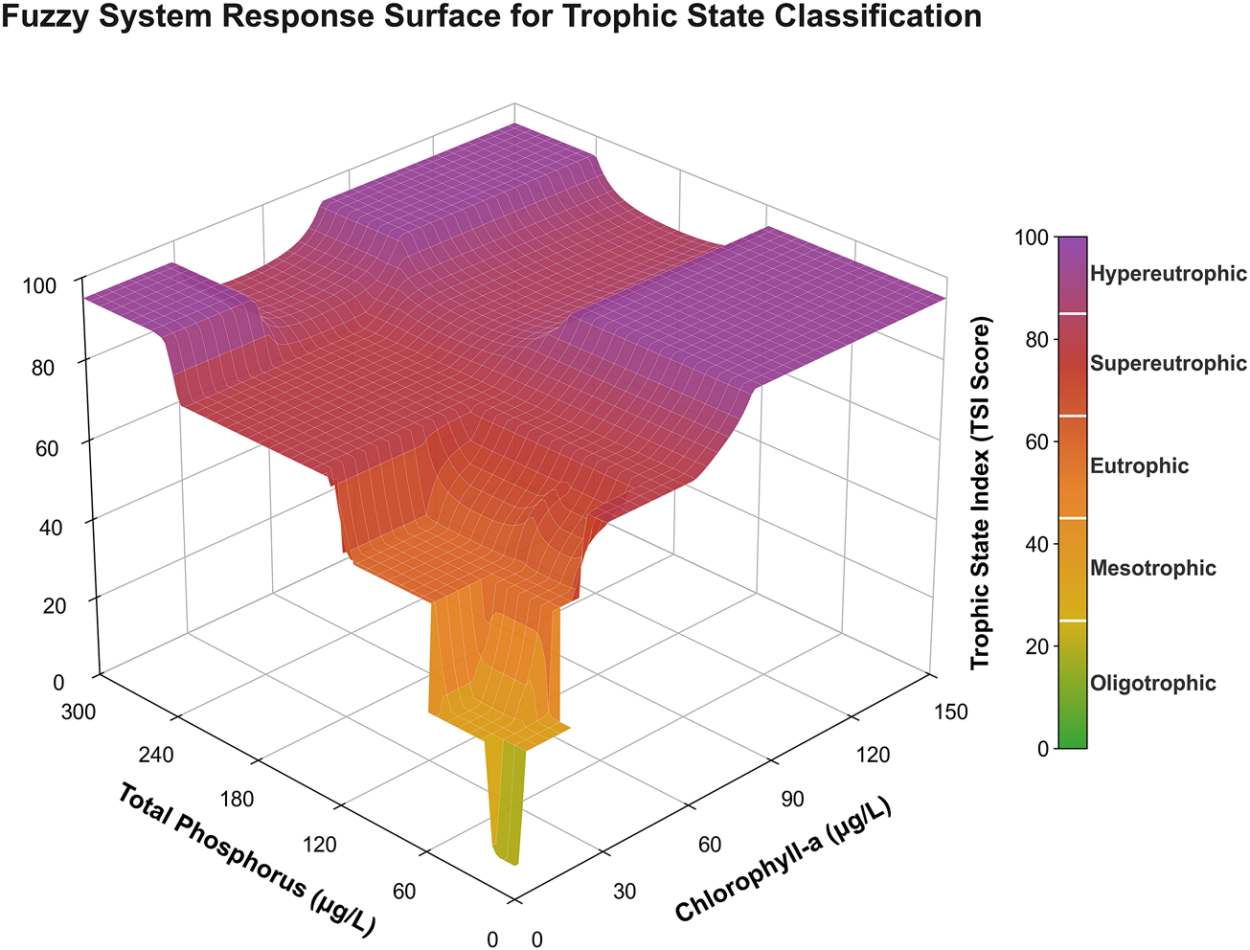
Three-dimensional response surface of the FIS for trophic state classification defining the ecological boundaries

Figure 7 displays the resulting response surface, organized dimensionally with Chl-*a*on the horizontal axis, TP on the depth axis, and the vertical elevation corresponding to the defuzzified *TSI Score*. Geometrically, this topography directly translates the logical interactions defined by the Mamdani IF-THEN rule base into a continuous classification spectrum. To facilitate the immediate visual identification of high-risk ecological regions, the surface was mapped onto a chromatic scale that transitions from a green basal plane (oligotrophic areas) to a purple elevated peak (hypereutrophic zones). Crucially, the surface exhibits smooth topographical gradients throughout the transitional boundaries between these classes. While single-parameter indexing frameworks often force discrete and mutually exclusive trophic categorizations that ignore marginal variation and multi-variable interactions (Gholizadeh et al. 2016), the smooth gradations empirically demonstrated in our model reflect the diffuse uncertainty inherent in limnological transitions, directly substantiating the fuzzy logic formulation of environmental indices (Gharibi et al. 2012; Yang et al. 2021, 2026). Furthermore, this geometrical modeling reveals a key system dynamic: although an isolated surge in either input variable is sufficient to significantly escalate the *TSI Score*, only the simultaneous occurrence of high concentrations in both parameters predictably triggers the system’s absolute maximum distress response.

**Fig. 8:**
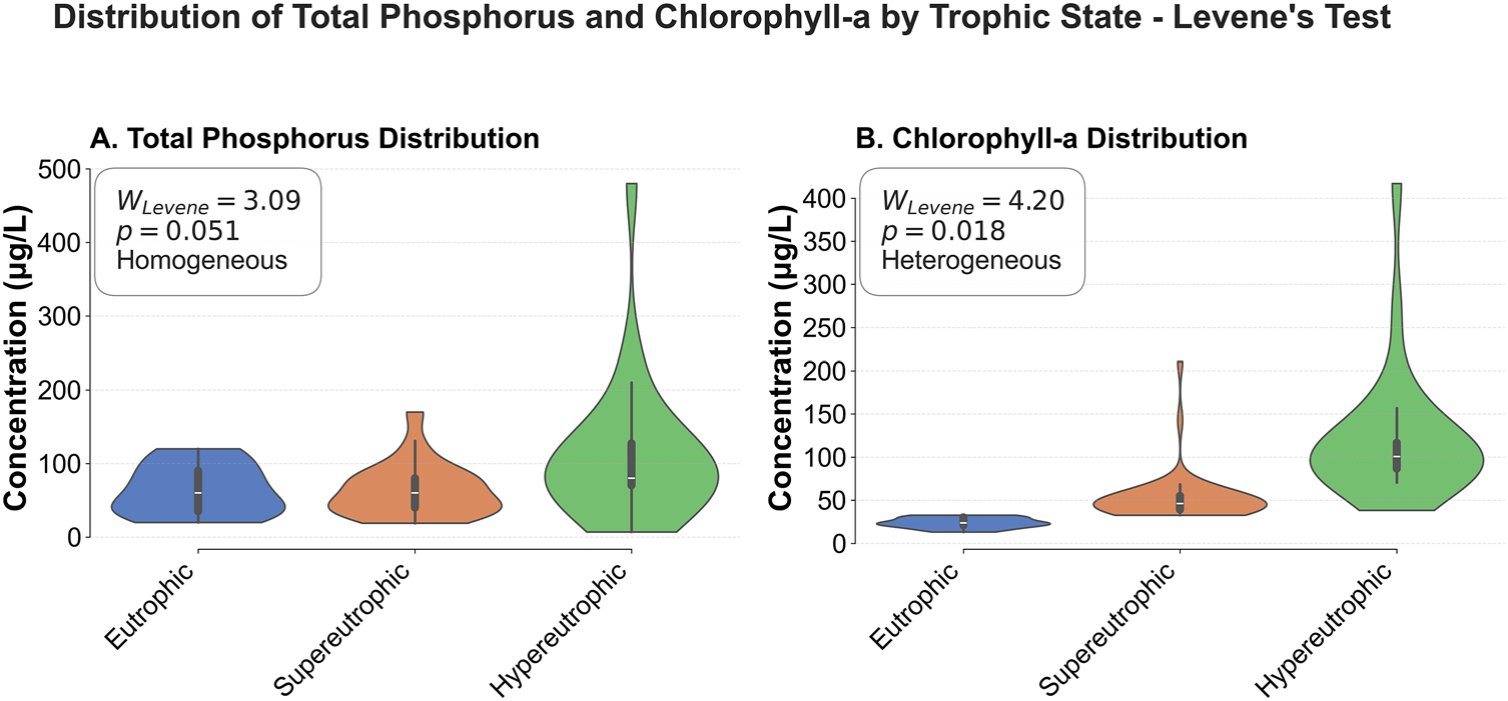
Distribution and variance analysis (Levene’s test) of TP and Chl-*a* concentrations across fuzzy trophic classes

### Ecological dynamics and variance analysis

Beyond global classification, understanding the structural dispersion of data across the trophic gradient is ecologically vital. The application of Levene’s Test (Fig. 8) revealed a substantial physiological dichotomy between the eutrophication driver and the biological response (Huisman et al. 2018; Burkholder and Glibert 2024). For TP, the statistically homogeneous variance maintained across the trophic spectrum signals a stable, perennial, and diffuse pressure on the aquatic system irrespective of the local degradation severity (Yuan et al. 2018). This homogeneity reflects the physical nature of phosphorus, which tends to disperse evenly throughout the water column, acting as a baseline driver for eutrophication (Yuan et al. 2018; Burkholder and Glibert 2024). Conversely, Chl-*a* demonstrated drastic heteroscedasticity, a direct consequence of the biological behavior of phytoplankton. Under favorable conditions, mobile cyanobacteria rapidly aggregate and form highly irregular surface scums (patchiness), leading to extreme spatial heterogeneity and variance explosion in advanced trophic states (Huisman et al. 2018; Soares et al. 2012). As corroborated by Qian et al. (2024), hypertrophic environments transition into chaotic responsive states, where abrupt and unpredictable algal blooms become a distinctive behavioral phenomenon rather than merely an elevation in global concentration means.

**Fig. 9:**
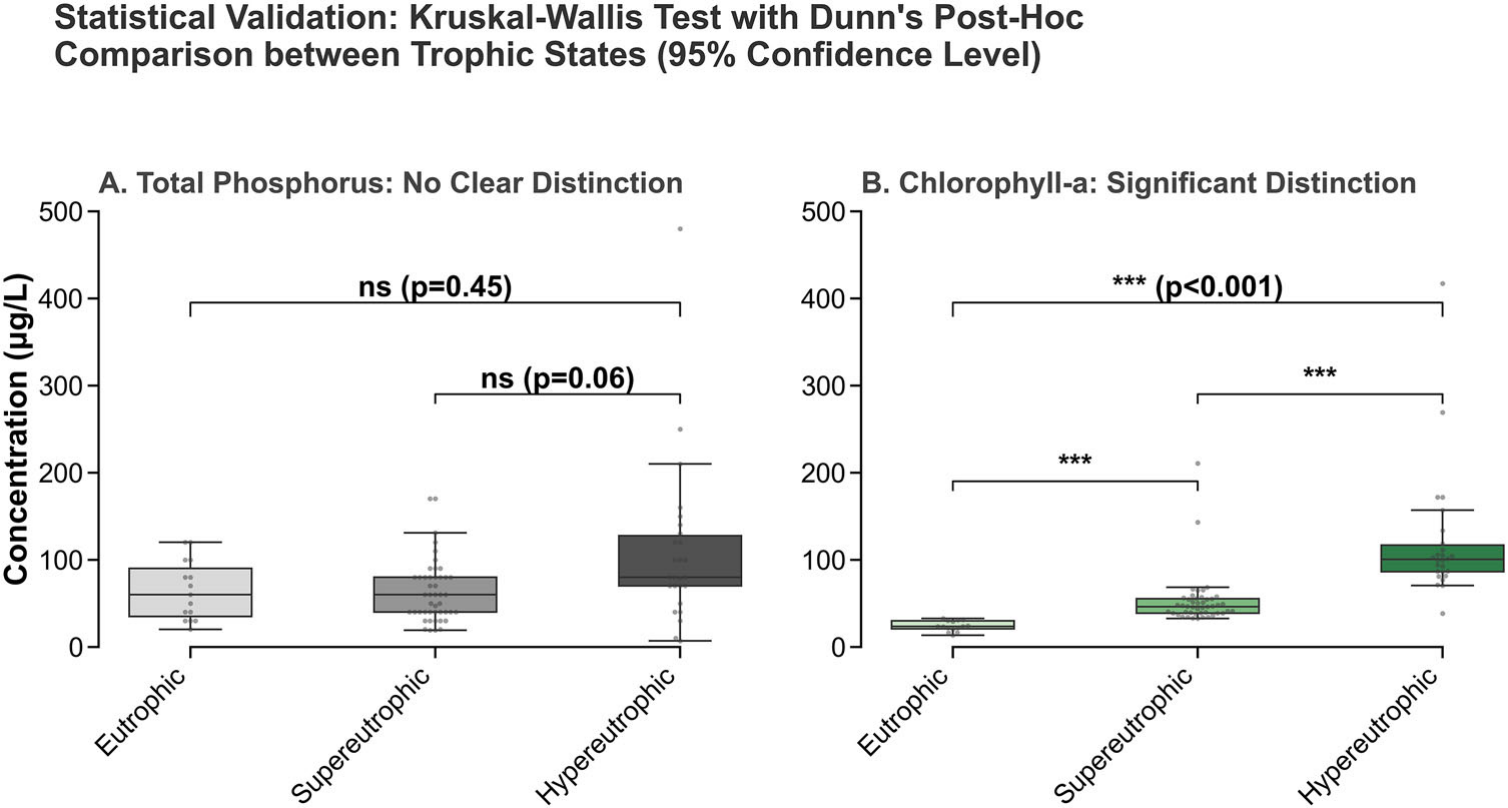
Dunn’s post hoc multiple comparison test for TP and Chl-*a* across different trophic classifications

Following the confirmation of heteroscedasticity by Levene’s test, which acted as a methodological gateway, Dunn’s post hoc pairwise multiple comparisons were employed to refine the statistical diagnosis across specific trophic intersections (Gilbert et al. 2024). This analysis consolidated the discriminatory capacity of the fuzzy criterion. Regarding Chl-*a*levels, the test confirmed highly significant mathematical distinctions across all paired configurations, demonstrating that phytoplankton communities remodel drastically at each trophic tier (Huisman et al. 2018; Soares et al. 2012). In stark contrast, Dunn’s investigation of TP attested to the absolute absence of inter-scalar differentiation between upper trophic boundaries. This scientifically endorses the premise that phosphorus compounds operate as a constant, structurally homogeneous diffuse pressure across all segments of the saturated reservoir, revealing the systemic flatness of TP (Fig. 9).

**Fig. 10:**
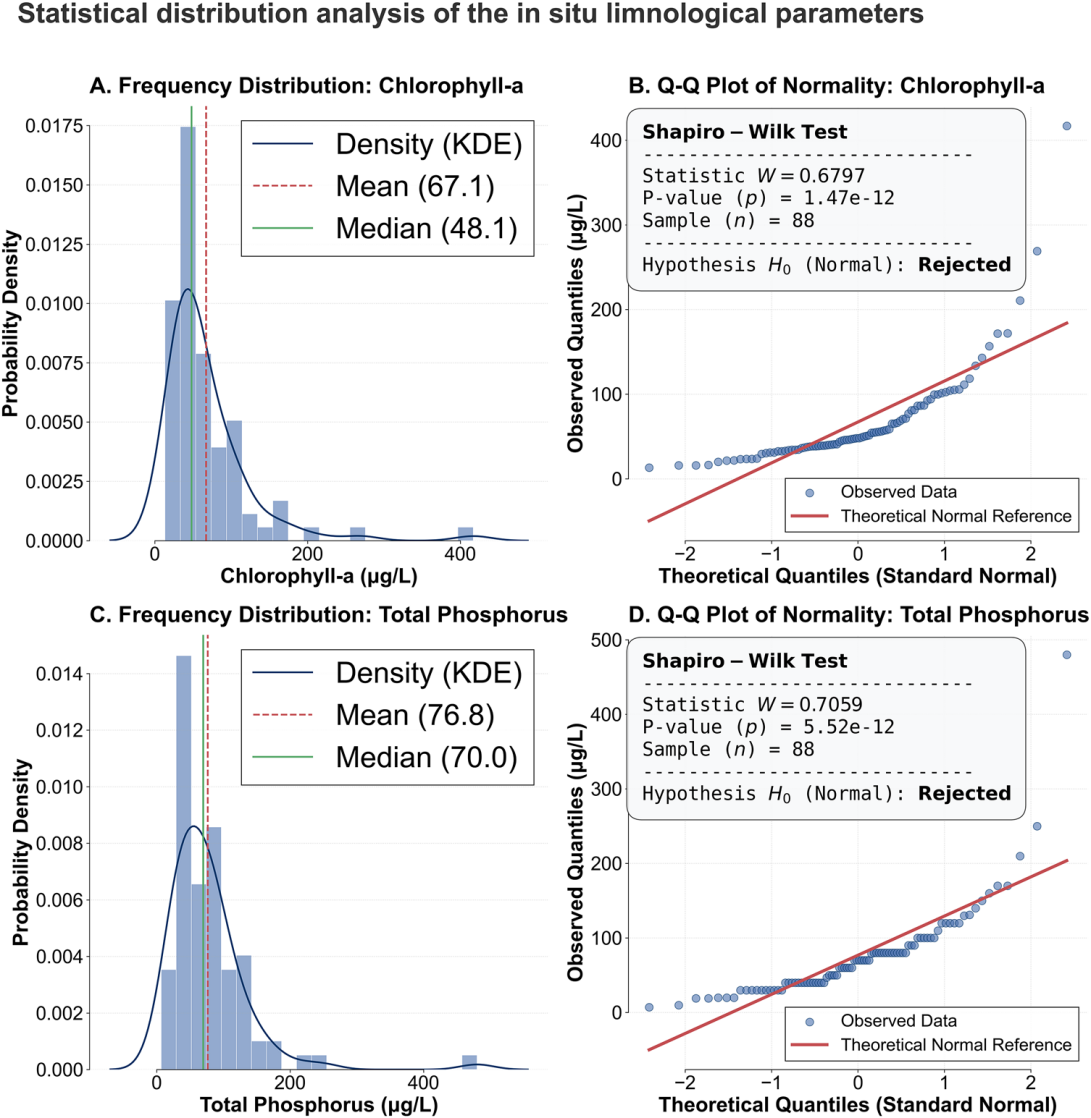
Statistical distribution analysis of the in situ limnological parameters: **A** frequency distribution of Chl-*a*, **B** Q-Q plot of normality for Chl-*a*, **C** frequency distribution of TP, and **D** Q-Q plot of normality for TP, graphically demonstrating the systematic departures from normality

**Fig. 11:**
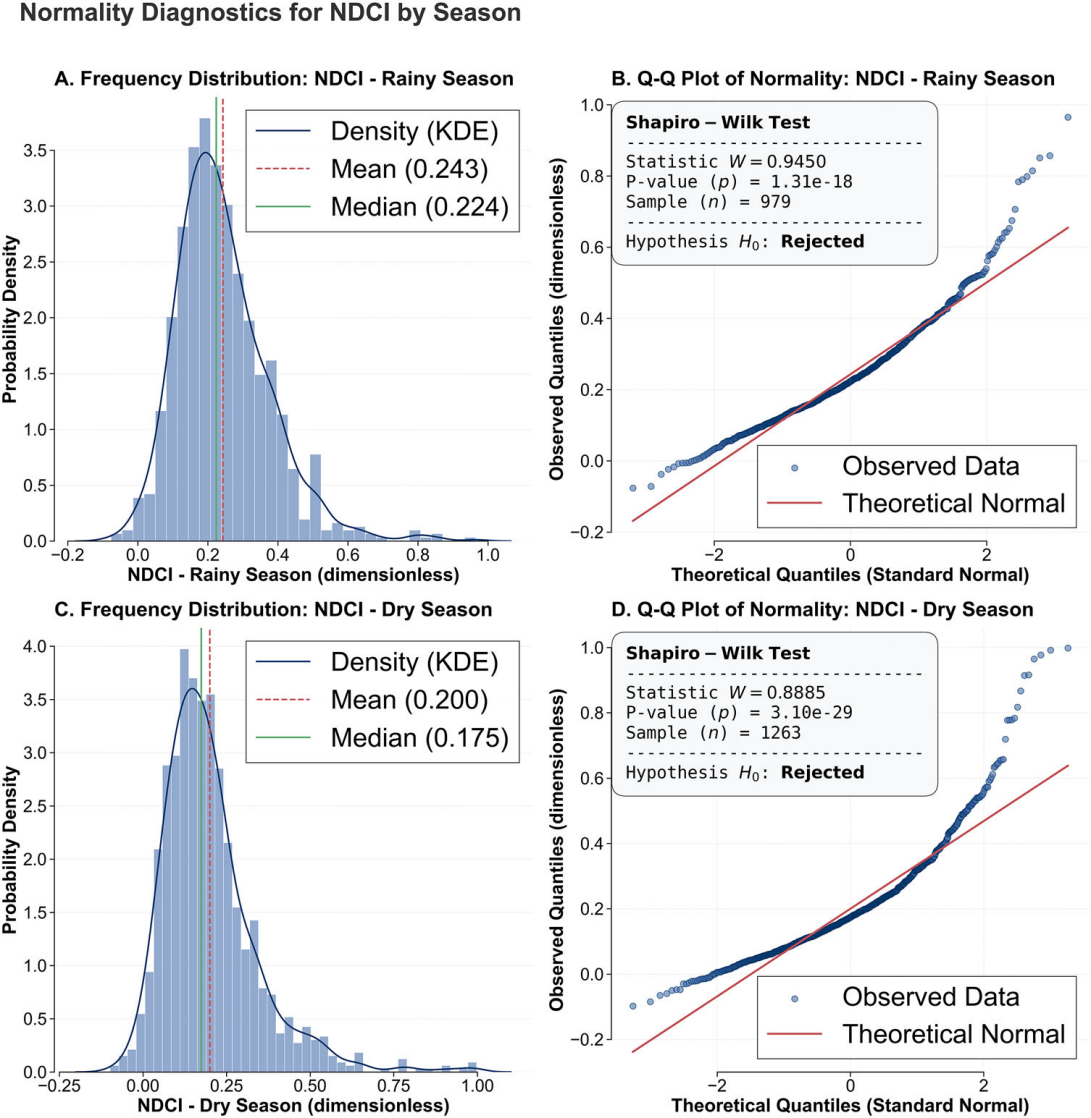
Structural evaluation of NDCI data distribution across distinct hydrological seasons: **A** frequency distribution and **B** corresponding Q-Q plot for the rainy season; **C** frequency distribution and **D** Q-Q plot for the dry season. The highly significant statistical parameters (p<0.001) strongly indicate the non-parametric nature of the retrieved orbital signal

**Fig. 12:**
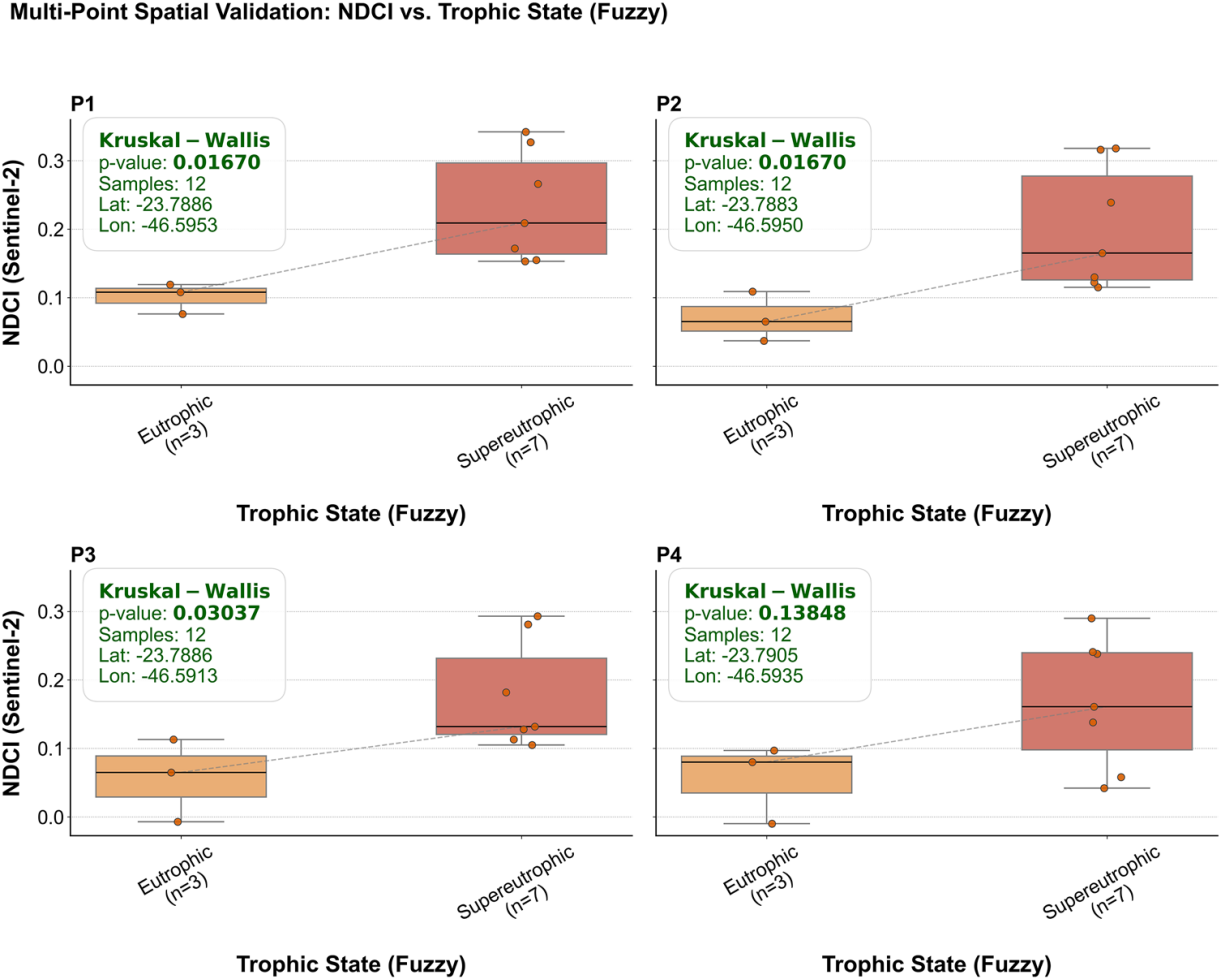
Multi-point spatial validation of the NDCI–trophic state relationship in the Billings Reservoir. Each panel (P1–P4) displays color-coded boxplots with jittered individual observations for the fuzzy-classified trophic categories, median-connecting dashed lines, and Kruskal-Wallis test statistics. Three of the four coordinates yield statistically significant results (p<0.05), while P4 (p=0.138) illustrates the spatial sensitivity boundary of the orbital validation

The in situ limnological data exhibited pronounced non-Gaussian behavior. Figure 10 illustrates this systematic departure from the theoretical normal distribution line for Chl-*a*and TP, aligning with the frameworks established by Bellini et al. (2025). Detailed statistical quantifications (Shapiro-Wilk *W* scores) for both in situ and orbital datasets are provided in the Material (Section S1). This severe deviation visually corroborates the Shapiro-Wilk test results, mathematically necessitating the abandonment of traditional parametric approaches (e.g., ANOVA) in favor of the Fuzzy logic framework and the non-parametric Kruskal-Wallis validation.

This non-Gaussian behavior was equally conserved in the orbital perspective: the NDCI data, stratified by season, exhibited structurally analogous deviations (Fig. 11), corroborating Mishra and Mishra (2012) and Jang et al. (2022).

### Spatio-temporal NDCI validation and long-term trophic evolution

A key challenge in cross-validating orbital indices against in situ classifications is the spatial uncertainty of conventional monitoring programs (Adjovu et al. 2023). The CETESB monitoring protocol reports a single nominal geographic coordinate for its sampling station; however, the actual acquisition coordinates may exhibit positional variability across campaigns due to vessel drift, GPS precision limitations, and the practical impossibility of revisiting an identical water parcel over a 16-year monitoring horizon. Conversely, the Sentinel-2 MSI sensor retrieves spectrally resolved reflectances at a native spatial resolution of 10–20 m, affording substantially higher geolocation precision (ESA 2024). The underlying premise is that the fuzzy-derived trophic classification remains representative across this confined spatial domain (Gharibi et al. 2012; Bellini et al. 2025), while the NDCI may exhibit subtle but ecologically meaningful variations attributable to sub-pixel heterogeneity, localized biomass gradients, and differential proximity to reservoir margins (Watanabe et al. 2018).

For each coordinate, the Kruskal-Wallis rank sum test was applied to evaluate whether NDCI distributions, whose theoretical basis was established by Mishra and Mishra (2012), differ significantly across the Fuzzy-classified trophic categories. This classification leverages the mathematical concepts of fuzzy logic (Gharibi et al. 2012; Yang et al. 2026), which were robustly adapted for water quality modeling by Gharibi et al. (2012). This non-parametric omnibus test was selected precisely because it requires neither normality nor homoscedasticity assumptions, prerequisites previously demonstrated to be violated by the NDCI data. The null hypothesis ($H_0$) postulates equal NDCI medians across all trophic classes; its rejection at α=0.05 establishes statistical evidence that the spectral index varies systematically as a function of the trophic state, an ecological premise documented in eutrophication studies (Gholizadeh et al. 2016; Huisman et al. 2018). Figure 12 presents the validation results as a 2×2 panel. Each subplot displays color-coded boxplots that visually translate the medians and interquartile ranges evaluated by the Kruskal-Wallis algorithm, overlaid with individual jittered observations for the trophic classes detected at the respective coordinate, accompanied by a dashed line connecting class medians to visually emphasize the index’s ascending monotonic trend with increasing trophy. At coordinates P1, P2, and P3, the Kruskal-Wallis test yielded highly significant *p*-values (p<0.05), confirming that NDCI distributions are statistically distinguishable across trophic categories and thereby validating the methodological premise that satellite-detectable spectral variations faithfully reflect compositional shifts in phytoplankton biomass.

Notably, the fourth coordinate (P4, p=0.138) returned a non-significant result, underscoring an important limitation of this approach: the NDCI-FIS relationship cannot be assumed as uniformly valid across all spatial domains of the reservoir. Because P4 is positioned at a greater distance from the nominal CETESB sampling station and closer to the margins, its spectral signal is likely confounded by sub-pixel heterogeneity, land-water adjacency effects, and localized hydrodynamic variations that decouple the orbital observation from the in situ reference point. Consequently, this non-significant outcome explicitly delineates the spatial boundary of our validation, indicating that the framework’s reliability decreases in near-shore or optically complex pixels, which reinforces the critical necessity for precise coordinate co-registration in multi-source environmental assessments (Pompêo et al. 2021).

**Fig. 13:**
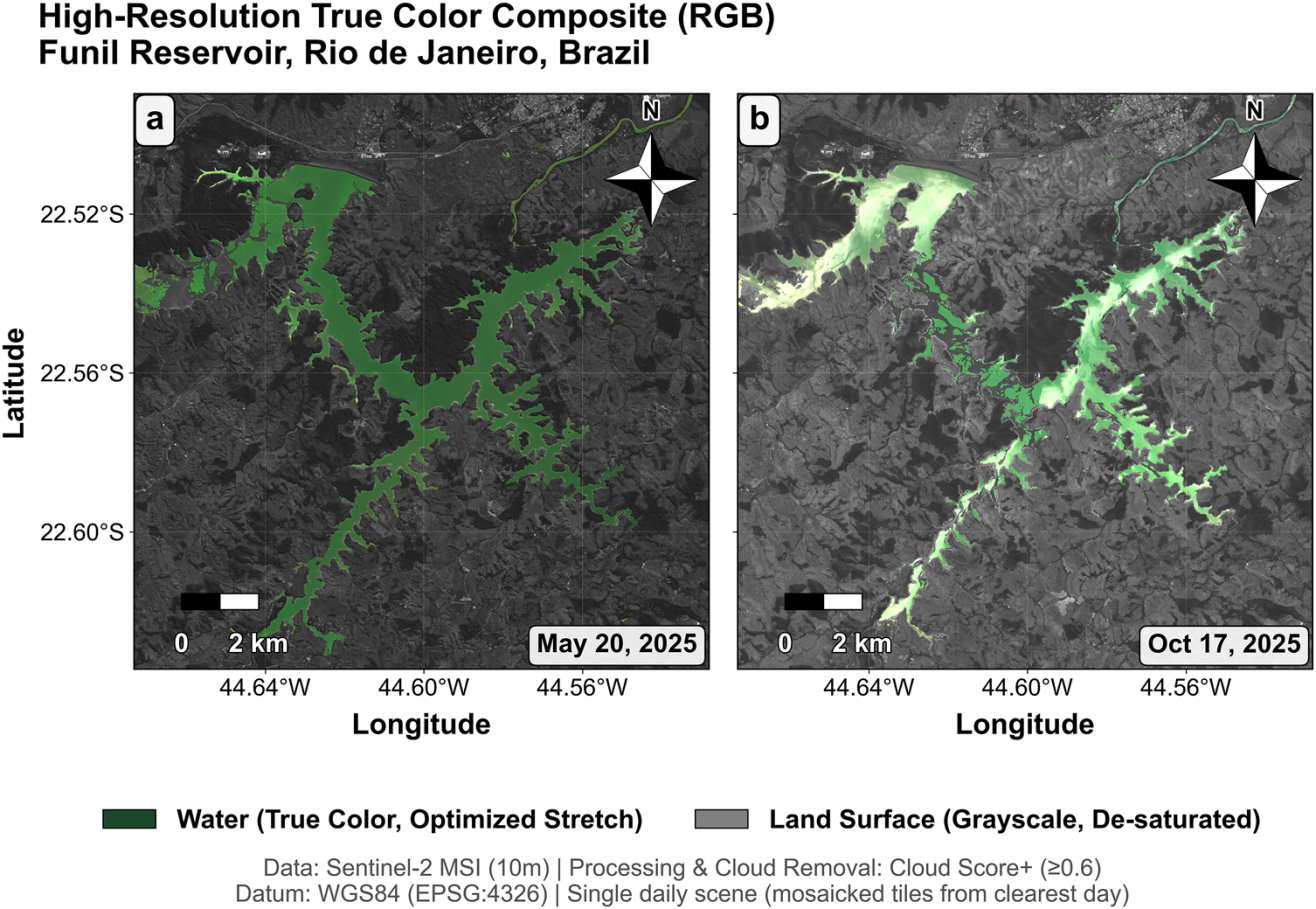
High-resolution true-color (RGB) composite of the Funil Reservoir illustrating seasonal optical variations. **a** captures the limnological conditions during the autumn period (May 2025), while **b** depicts the spring continuum (October 2025). The adjacent terrestrial matrix is rendered in grayscale to exclusively highlight the chromatic signature of the water body. Data acquired via the Sentinel-2 (MSI) sensor natively at 10 m resolution and processed computationally through GEE using Cloud Score+ (≥0.60) filtering

### Application case: spatial and seasonal dynamics in the Funil Reservoir

Having established the statistical robustness of the NDCI against historical in situ trophic classifications at the Billings Complex, the methodological framework was consequently extrapolated to the Funil Reservoir to evaluate its operational scalability and spatial analytical capacity, an approach previously validated by Watanabe et al. (2018). The Funil impoundment operates under a highly dynamic hydrological regime, where seasonal precipitation governs not only the influx of allochthonous nutrients from the heavily impacted Paraíba do Sul watershed, but also the hydraulic residence time, a critical controlling factor for cyanobacterial development demonstrated by Soares et al. (2012). To characterize these pronounced intra-annual disparities and demonstrate the synoptic capabilities of modern orbital remote sensing, we capitalized on the petabyte-scale cloud-computing architecture of GEE (Gorelick et al. 2017). By systematically processing native 10-m spatial resolution data from the *Sentinel-2* MSI and applying the *Cloud Score+* algorithm for rigorous atmospheric masking, we successfully isolated optically clear scenes irrespective of the region’s frequent cirrus and aerosol interference.

Figure 13 presents a seamless, algorithmically optimized true-color (RGB) composite comparing the reservoir’s limnological surface characteristics between the contrasting transition periods of May and October 2025, acting as a qualitative preliminary step to visually confirm large-scale optical differences prior to quantitative spectral extraction. This high-fidelity visualization employs a dual-rendering technique (masking the terrestrial background in de-saturated grayscale while applying optimized radiometric stretching to the aquatic matrix) to drastically isolate and enhance the intrinsic apparent optical properties of the water column, utilizing the optical principles of cyanobacteria scattering and absorption described by Zhang et al. (2018). The conspicuous chromatic divergences observable between the temporal panels serve as a macroscopic, qualitative proxy for significant shifts in water composition. As documented by Shi et al. (2019) and Watanabe et al. (2018), these elemental color variations structurally reflect seasonal fluctuations in optically active components, such as suspended inorganic matter and aggregate phytoplankton biomass. Consequently, this visual characterization provides a crucial geospatial context preceding the quantitative derivation of the spectral eutrophication indices, aligning with the theoretical foundations of Mishra and Mishra (2012) and recent applications by Zheng et al. (2024).

**Fig. 14:**
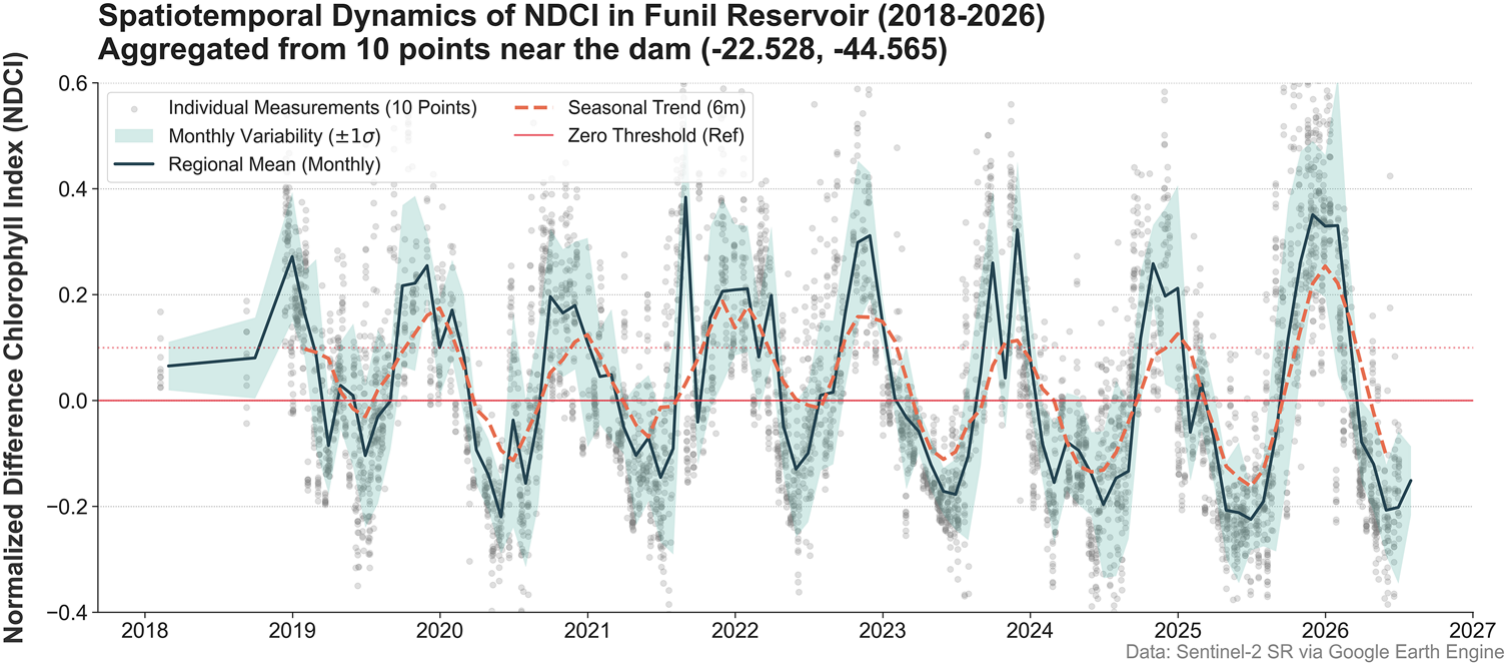
Spatiotemporal dynamics of the NDCI in the Funil Reservoir from 2018 to 2025. Data points representing individual measurements from ten spatially distributed coordinates near the reservoir dam are overlaid with the aggregated monthly regional mean (solid line) and its corresponding standard deviation envelope ($\pm 1\sigma$). A 6-month moving average (dashed line) delineates the long-term seasonal trend. The spectral retrieval incorporated rigorous atmospheric filtering (Cloud Score+) and sun-glint correction methodologies

**Fig. 15:**
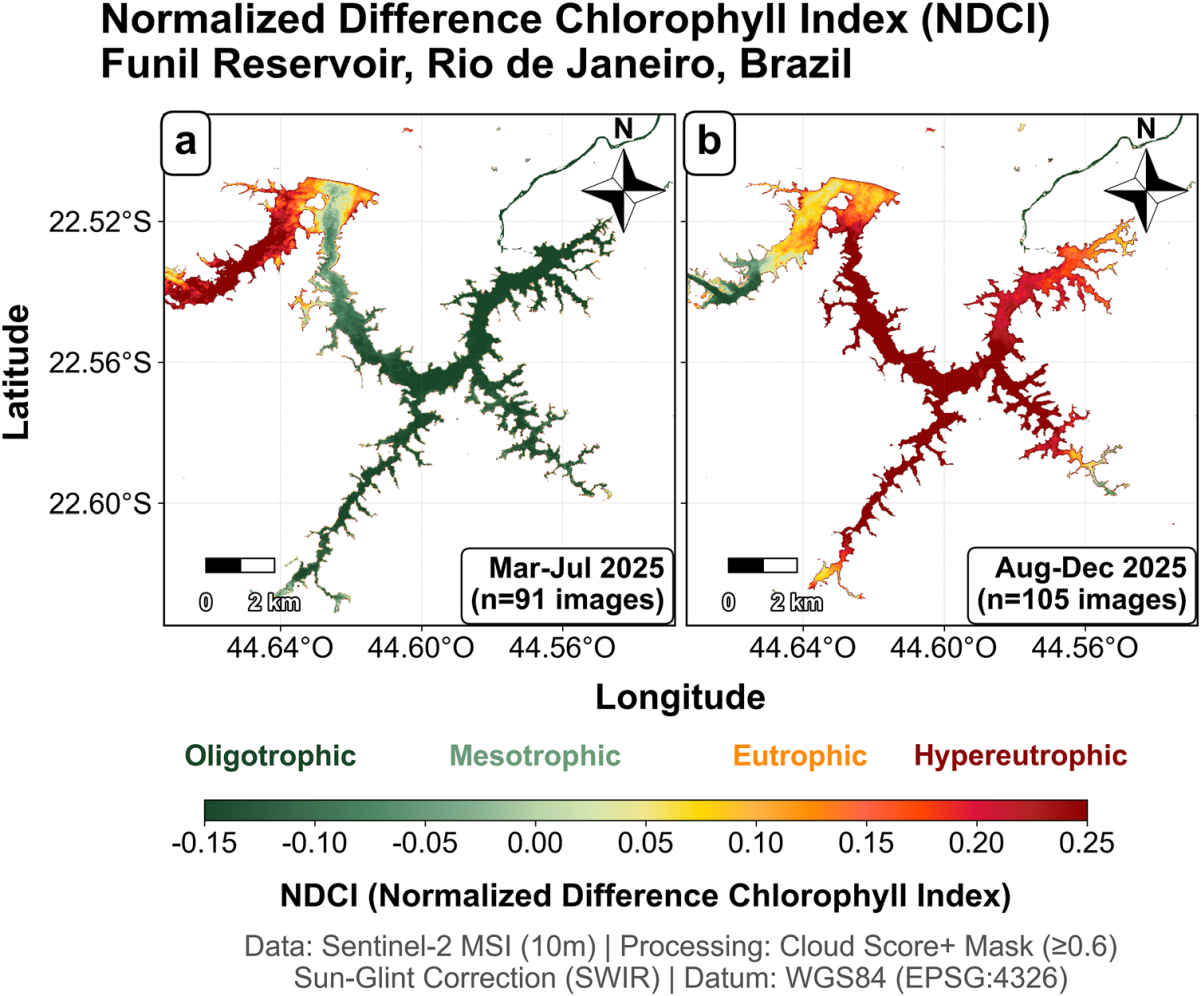
Comparative spatial mapping of trophic states in the Funil Reservoir based on the NDCI. The categorization thresholds are adapted from the bio-optical modeling established by Mishra and Mishra (2012)

**Fig. 16:**
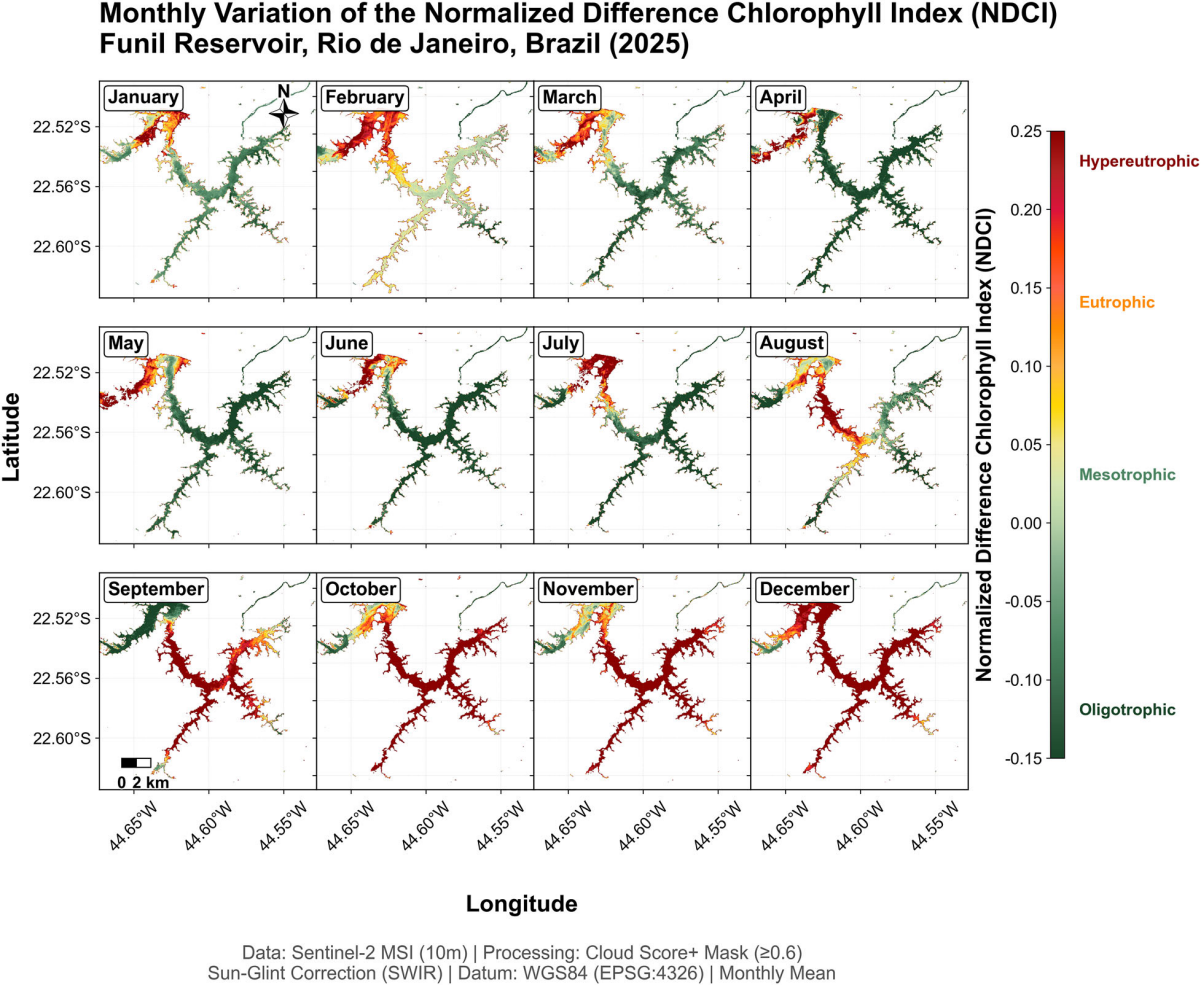
Monthly synoptic spatiotemporal panels tracking the spatial variation of the NDCI and corresponding trophic states across the Funil Reservoir throughout 2025. Categorizations are anchored on the Mishra and Mishra (2012) thresholds. The data exposes both the seasonal progression of algal blooms and the spatial vulnerability of distinct morphological compartments within the reservoir

Building upon this qualitative overview, a quantitative longitudinal assessment of phytoplankton biomass was conducted by retrieving the NDCI from 2018 to 2025. To ensure statistical representativeness and mitigate localized hydrodynamic anomalies, spectral data were systematically extracted from a spatial cluster of ten discrete equidistant points positioned adjacent to the reservoir’s principal dam (spanning approximately from −22.527 to −22.530$^\circ$ latitude and −44.562 to −44.567$^\circ$ longitude). This multispatial extraction strategy (Bresciani et al. 2020), governed by an automated *GEE* pipeline (Tamiminia et al. 2020), incorporated the *Cloud Score+* (threshold ≥0.60) masking protocol alongside shortwave-infrared (B12) subtraction for sun-glint correction.

The ensuing multi-year time series (Fig. 14) elucidates the spatiotemporal dynamics of the trophic state with high granular resolution. The plotted standard deviation envelope ($\pm 1\sigma$) mathematically captures the high intrinsic spatial heterogeneity across the reservoir’s surface during each monthly retrieval. While Bresciani et al. (2020) highlighted the critical necessity of modern optical sensors to capture fine-scale phytoplankton variations, our aggregation of individual point measurements into a monthly regional mean successfully uncovers a pronounced seasonal periodicity, superimposed upon a baseline indicative of sustained eutrophic pressure. Furthermore, while the 6-month moving average robustly characterizes the long-term ecological trajectory, the scatter of individual satellite observations reveals substantial intra-monthly spatial variability within the monitored sector, a structural heterogeneity inherent to dendritic tropical reservoirs. Notably, the recurrent cyclical peaks crossing the zero-threshold baseline, an established empirical boundary originally validated by Mishra and Mishra (2012) for elevated Chl-*a* concentrations, quantitatively track the temporal inception and duration of major algal bloom events. Ultimately, these quantitative results strongly corroborate the classical associations described by Paerl and Huisman (2008) and the contemporary findings of Oliver et al. (2020), who demonstrated the acute vulnerability of continuous water bodies to synergistic seasonal environmental triggers governing algal dynamics.

To capture the reservoir’s full spatial heterogeneity (a methodological imperative frequently emphasized in tropical limnological assessments by Wengrat and de Campos (2011)), we translated the NDCI continuous values into a synoptic categorical map of trophic states. The classification thresholds were rigorously grounded in the seminal framework proposed by Mishra and Mishra (2012), who originally formulated the NDCI for turbid, productive estuarine and coastal waters. Following their empirical demonstration that NDCI variations strongly correlate with absolute Chl-*a*concentrations (ranging from oligotrophic baselines to severe algal blooms), we established discrete intervals to categorize the water body from oligotrophic (NDCI <-0.05) to hypereutrophic (NDCI >0.22). This geospatial categorization strategy is supported by the remote sensing applications described by Pizani et al. (2020), who validated the efficacy of such spectral compartmentalization in complex, optically active inland and transitional waters. Figure 15 illustrates the direct application of this categorical framework, demonstrating how the continuous orbital signal is operationalized into discrete management zones. It presents a comparative spatial snapshot that corroborates the spectral distinctions previously observed in the true-color composite.

The ultimate scalability of this orbital framework is illustrated through the generation of high-resolution temporal panels, building on the geo-big data concepts extensively reviewed by Tamiminia et al. (2020). Figure 16 presents a panoramic monthly tracking of the Funil Reservoir throughout the year of 2025. This 2×6 mosaic visually documents the geographical expansion and contraction of algal blooms across the reservoir’s dendritic morphology, highlighting critical zones of chronic eutrophication, particularly in the shallower, lentic branches susceptible to prolonged hydraulic retention. This structural vulnerability and spatial heterogeneity align perfectly with the limnological dynamics previously characterized by Soares et al. (2012). By synthesizing millions of orbital data points into an intuitive, color-coded trophic diagnostic, this approach effectively bridges the gap between complex bio-optical physics and practical environmental governance. Ultimately, the ability to comprehensively map the entire water body on a monthly cadence proves that the synergistic use of the Sentinel-2 constellation and state-of-the-art cloud computing can readily upgrade reservoir surveillance from a reactive constraint to a proactive, predictive management paradigm, a technological transition increasingly demanded by modern monitoring frameworks (Acuña-Alonso et al. 2025).

## Conclusion

This study demonstrated that the fusion of fuzzy logic trophic classification with NDCI-based orbital remote sensing constitutes a statistically robust, spatially scalable framework for continuous reservoir monitoring. The 16-year cross-validation against the Billings Reservoir’s limnological record confirmed NDCI as a high-fidelity proxy for trophic state classification in optically complex Case 2 waters, while the application to the Funil Reservoir illustrated the methodology’s potential transferability to morphologically distinct, data-scarce systems. The spatiotemporal mosaics revealed the geographic expansion and contraction of blooms at monthly cadences, a synoptic resolution fundamentally unattainable through conventional point-wise in situ campaigns.

The synergistic application of cloud computing architectures and open-access satellite constellations marks a significant advancement in environmental governance. Traditional water quality management has historically been a reactive endeavor, reliant on sparse datasets that often fail to detect transient, high-risk biomass peaks. In contrast, our proposed framework establishes a scalable observational tool that could support future early-warning mechanisms capable of near real-time surveillance. If transitioned into an operational system, this proactive capability holds the potential to assist water treatment operators and public health officials, theoretically affording them the necessary lead time to implement strategic mitigation protocols before acute cyanotoxin exposure compromises urban water supplies or downstream ecological integrity.

Despite its high translational impact, this orbital methodology presents intrinsic structural challenges and boundaries yet to be completely overcome in remote sensing limnology. Specifically, the NDCI-FIS framework acts purely as a proxy for total phytoplankton biomass and cannot isolate the specific risks of toxic cyanobacteria. While recent studies with modern satellites have demonstrated capabilities in recognizing cyanobacterial species in oceans, achieving species-level differentiation remains a distant reality in inland (Case 2) waters. Inferring the cyanobacteria phylum fundamentally depends on massive amounts of local data, and the severe scarcity of concurrent in situ datasets restricts the ability to successfully train and scale modern machine learning algorithms for taxonomic proxy classification. Therefore, our study allows us to infer chlorophyll-*a* biomass as a viable proxy for algal blooms, but without direct taxonomic discrimination. Future research must prioritize the expansion of high-frequency in situ telemetry networks to supply the data volume demanded by deep learning architectures.

Ultimately, while the fusion of remote sensing and fuzzy logic represents a methodological advancement, algorithmic sophistication alone cannot replace physical limnological infrastructure. The deployment of increasingly complex artificial intelligence models requires an uninterrupted flow of high-quality validation data to prevent calibration drift and algorithmic bias. Therefore, achieving predictive and mechanistic water security substantially depends on the unwavering commitment of governmental bodies to enact robust public policies. Expanding high-frequency in situ measurement networks and ensuring unconditional open-access to limnological data pipelines are necessary to safeguard the world’s most critical water resources in an era of climate acceleration.

## Data Availability

The historical limnological datasets utilized in this study are in the public domain and can be originally accessed through the *Infoaguas* system maintained by CETESB. To ensure total reproducibility, an exact replica of the compiled datasets employed in our analyses has been deposited and is freely accessible in a public repository under the DOI: https://doi.org/10.5281/zenodo.18365025.
